# Supplementary material for: The renal lineage factor PAX8 controls oncogenic signalling in kidney cancer
Source: Nature. 2022 Jun 8;606(7916):999–1006. doi: 10.1038/s41586-022-04809-8 (PMC9242860; doi:10.1038/s41586-022-04809-8)
Supplement: Supplementary file 1 — This file contains Supplementary Figs. 1–4 and Supplementary Methods. Supplementary Fig. 1: Uncropped immunoblots from Extended Data Figs. 1–7. Supplementary Fig. 2: Uncropped immunoblots from Extended Data Figs. 9 and 10. Supplementary Fig. 3: Full scans of EMSAs. Supplementary Fig. 4: Gating strategy for competition assay. [file 41586_2022_4809_MOESM1_ESM.pdf]

nature portfolio
https://doi.org/10.1038/s41586-022-04809-8

Supplementary information

The renal lineage factor PAX8 controls oncogenic signalling in kidney cancer

In the format provided by the authors and unedited

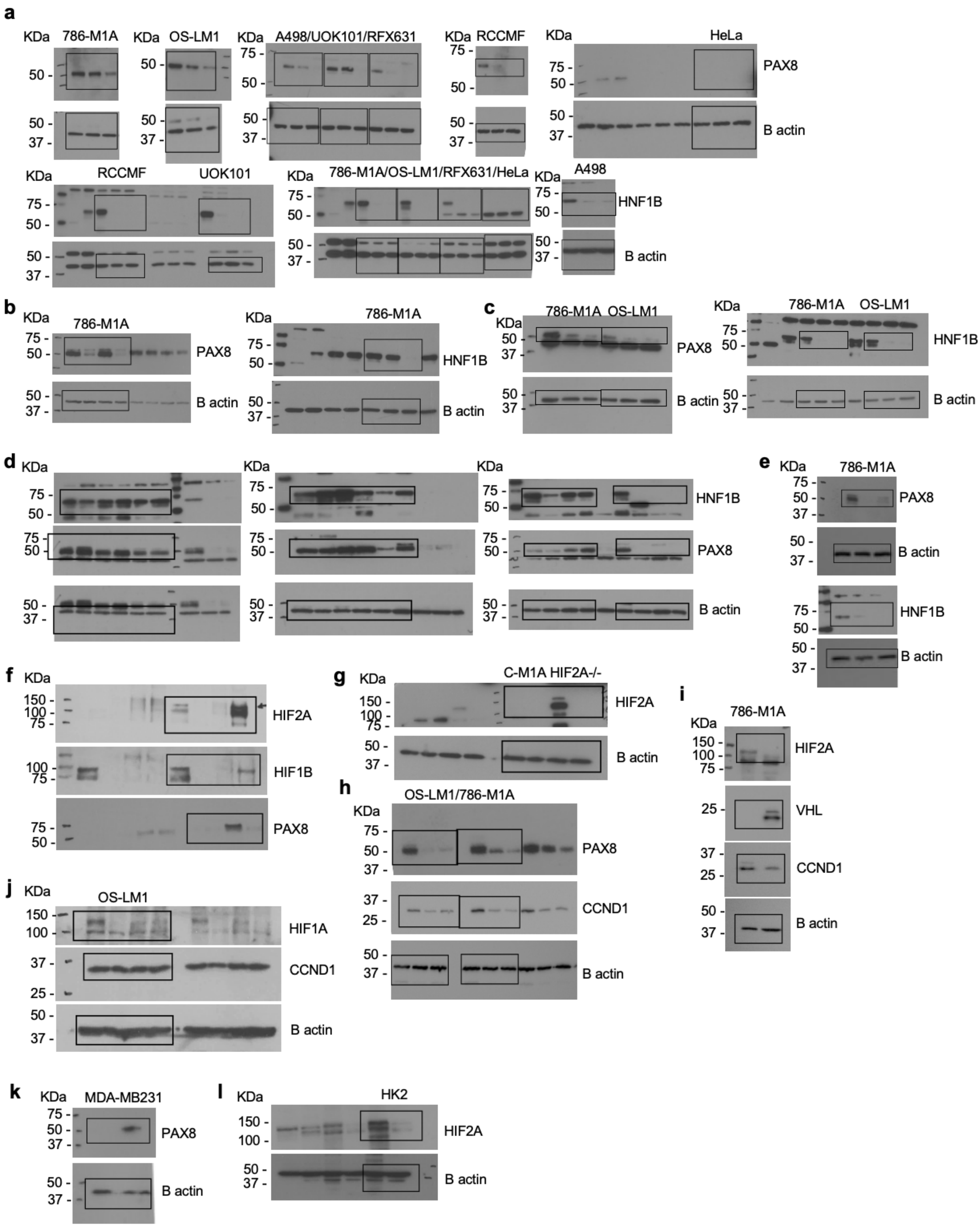
a
KDa
786-M1A
OS-LM1
A498/UOK101/RFX631
RCCMF
HeLa
PAX8
B actin
RCCMF
UOK101
786-M1A/OS-LM1/RFX631/HeLa
A498
HNF1B
B actin
b
786-M1A
PAX8
B actin
786-M1A
HNF1B
B actin
c
786-M1A OS-LM1
PAX8
B actin
786-M1A OS-LM1
HNF1B
B actin
d
HNF1B
PAX8
B actin
e
786-M1A
PAX8
B actin
HNF1B
B actin
f
HIF2A
HIF1B
PAX8
g
C-M1A HIF2A-/-
HIF2A
B actin
h
OS-LM1/786-M1A
PAX8
CCND1
B actin
i
786-M1A
HIF2A
VHL
CCND1
B actin
j
OS-LM1
HIF1A
CCND1
B actin
k
MDA-MB231
PAX8
B actin
l
HK2
HIF2A
B actin

Supplementary Figure 1 | Uncropped immunoblots from Extended Data Figures 1-7. a. Immunoblots from Extended Data Fig.1 c. **b.** Immunoblots from Extended Data Fig. 1f. **c.** Immunoblots from Extended Data Fig. 1g. **d.** Immunoblots from Extended Data Fig. 2c. **e.** Immunoblots from Extended Data Fig. 3b. **f.** Immunoblots from Extended Data Fig. 4g. **g.** Immunoblots from Extended Data Fig. 5a. **h.** Immunoblots from Extended Data Fig. 6d. **i.** Immunoblots from Extended Data Fig. 6g. **j.** Immunoblots from Extended Data Fig. 6j. **k.** Immunoblots from Extended Data Fig. 7b. **l.** Immunoblots from Extended Data Fig. 7d.

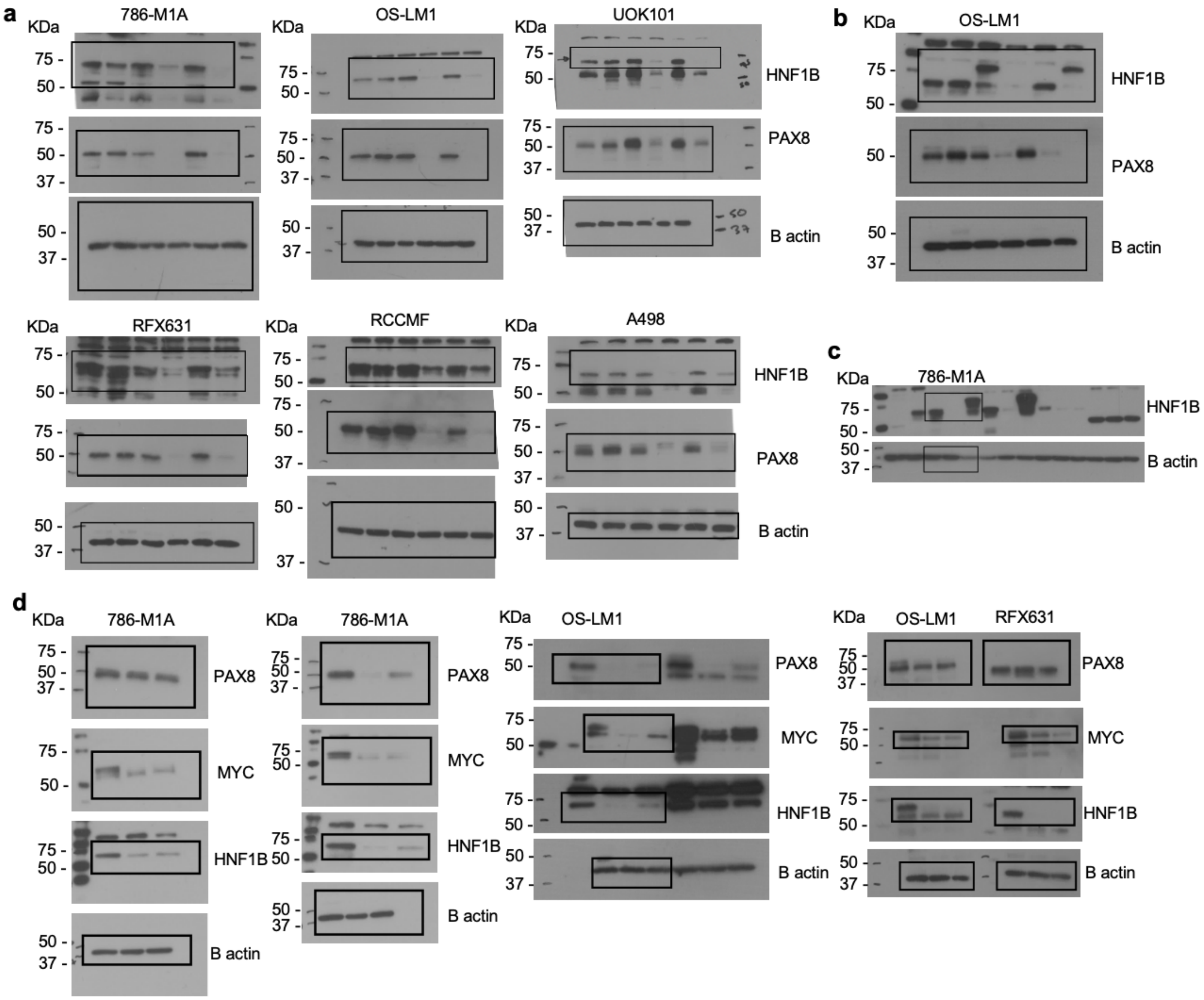

Supplementary Figure 2 | Uncropped immunoblots from Extended Data Figures 9-10. a. Immunoblots from Extended Data Fig. 9c. **b.** Immunoblots from Extended Data Fig. 9g. **c.** Immunoblots from Extended Data Fig. 9i. **d.** Immunoblots from Extended Data Fig. 10c-e.

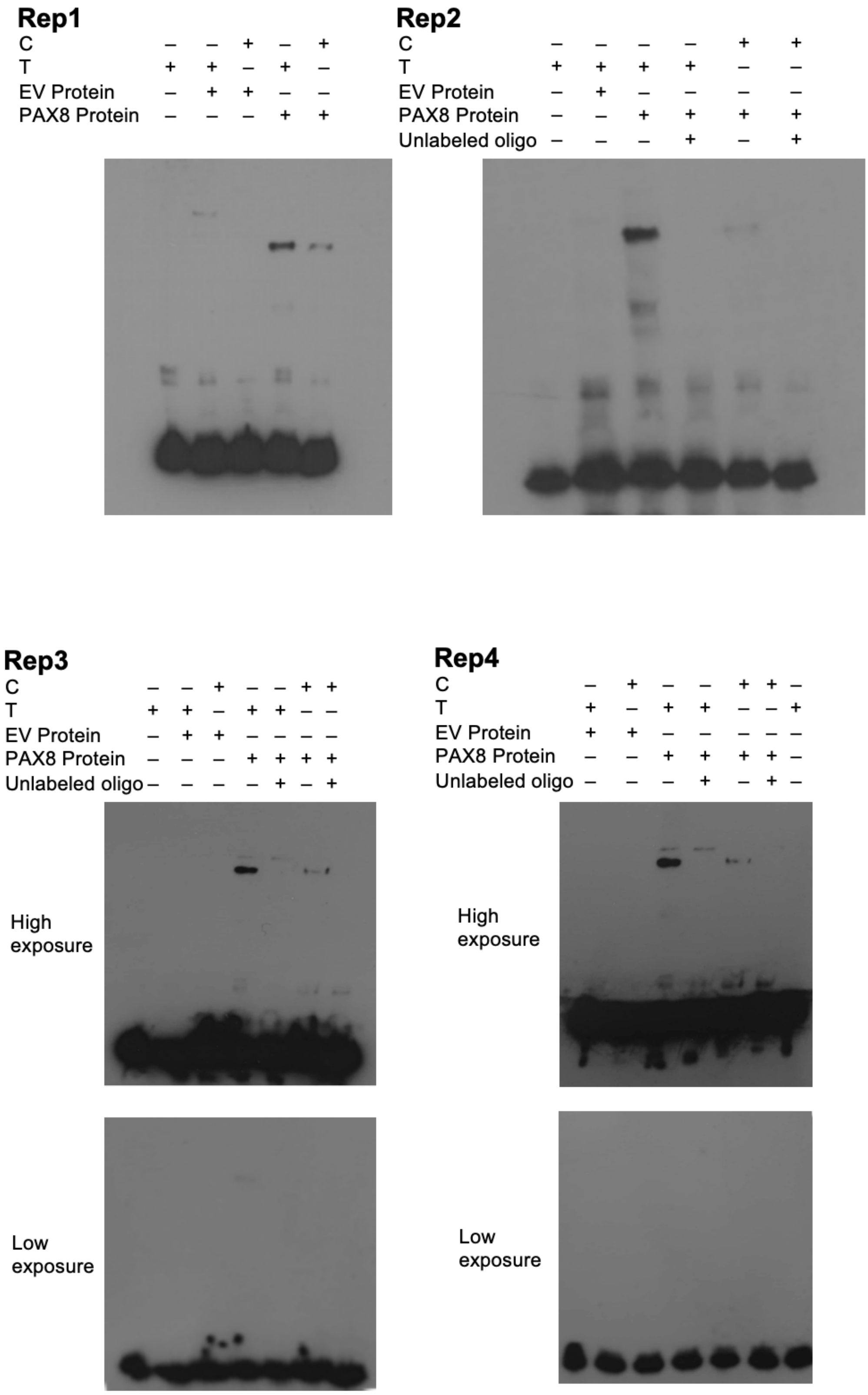

Supplementary Figure 3 | Full scans of EMSA replicates.

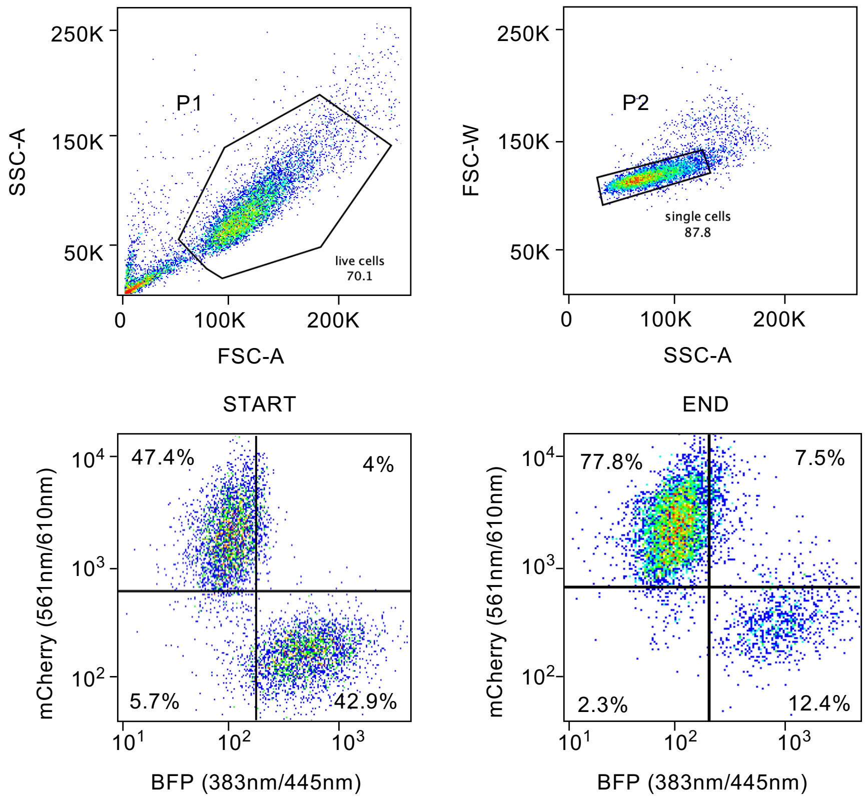

Supplementary Figure 4 | Gating strategy for competition assay.

Methods

Cell lines

The UOK101 cell line was obtained from M. Linehan (the UOB Tumor Cell Line Repository, National Cancer Institute, Bethesda, MD). The HK2 cell line was obtained from C. Frezza (MRC Cancer Unit, Cambridge, UK). ACHN and CAKI-2 were obtained from E. Maher (Department of Medical Genetics, University of Cambridge, UK). All other human cancer cell lines and HEK293T cells were obtained from J. Massagué (MSKCC, New York, USA). 786-M1A and OS-LM1 are the respective metastatic derivatives of 786-O and OS-RC2 cells and have been previously described[14]. 2801-LM1 is a metastatic derivative of 786-O and it does not carry a luciferase reporter gene. C-M1A$^{HIF2A-/-}$ is a single cell-derived HIF2A$^{-/-}$ clone from 786-M1A cells generated by CRISPR-Cas9 mediated knockout of HIF2A using sgEPAS1-5 (5'- TGAGATTGAGAAGAATGACG-3') in which HIF2A inactivation was validated by Sanger sequencing and Western blotting for HIF2A. Cell lines were authenticated by short-tandem repeat profiling. Mycoplasma negativity was confirmed by the MycoAlert^{TM} Mycoplasma Detection Kit (Lonza, LT07-318) or by qRT-PCR (PhoenixDx® Mycoplasma Mix). HEK293T cells were used for lentivirus production. RCC cell lines were cultured in RPMI-1640 medium (Sigma) supplemented with 10% FBS, penicillin (100U/mL) and streptomycin (100µg/mL). All other cell lines were cultured in DMEM medium (Invitrogen) supplemented with 10% FBS, penicillin (100U/mL) and streptomycin (100µg/mL). Cell lines were used in experiments within 15 passages from thawing.

Plasmids

psPAX2 and pMD2.G were gifts from Didier Trono (Addgene plasmid # 12260 and # 12259), pCW-Cas9 was a gift from Eric Lander & David Sabatini (Addgene plasmid # 50661)[43], lentiCas9-Blast was a gift from Feng Zhang (Addgene plasmid # 52962)[44], pHR-SFFV-KRAB-dCas9-P2A-mCherry was a gift from Jonathan Weissman (Addgene plasmid # 60954)[45], pKLV-U6gRNA(BbsI)-PGKpuro2ABFP (Addgene plasmid # 50946)[46] and pKLV2-U6gRNA5(BbsI)-PGKpuro2ABFP-W (Addgene plasmid # 67974)[47] were gifts from Kosuke Yusa. The latter was modified to generate the following plasmid variants: pKLV-U6-gRNA(BbsI)-PGKhygro2ABFP and pKLV-U6-gRNA(BbsI)-PGKhygro2AmCherry. DOX-inducible shRNA expression plasmids LT3REVIR and LT3GEPIR were kindly gifted by J. Zuber (IMP, Vienna)[48]. LT3GEPIR was modified to generate LT3GEBIR, where the puromycin resistance gene was replaced by BFP. For the reporter assays, STARR-seq luciferase validation vector_ORI_empty was a gift from Alexander Stark (Addgene plasmid # 99297)[49]. *PAX8* cDNA was amplified from pCMV3-PAX8 (HG14505-UT, Sino Biological). *HNF1B* cDNA was amplified from pLCMV:ECFP(loxP)(FRT)HNF1B, a gift from Gerhart Ryffel (Addgene plasmid # 31439)[50]. For constitutive expression, pLVX-Puro (632164, Clontech) was used to exogenously express the cDNA constructs. All sgRNA and shRNA constructs used in this study were purchased from Sigma-Aldrich and are listed in Supplementary Table 10.

Lentiviral production and transduction

HEK293T cells were transfected with a mixture of the lentiviral transfer plasmid of interest, psPAX2 and pMD2.G using FuGENE 6 transfection reagent (Promega E269A). The media containing the lentivirus was collected 48-72 hours post-transfection and filtered through a 0.45μM PVDG sterile filter. Cells were transduced

with the lentiviral supernatant in the presence of 6-8μg/mL Polybrene (Millipore). Puromycin (4μg/ml, Invivogen) or hygromycin (800μg/ml, Invivogen) selection started 24-48 hours post-transduction.

Transcription factor sgRNA library production

Genes encoding transcription factors were identified from a previously published list[51]. The library also contained 100 non-targeting control sgRNAs. The sequences for the sgRNAs were obtained from Wang et al.[43] with 7 sgRNAs selected for each gene (Supplementary Table 11). Oligos were ordered from Custom Array Inc. Oligos were amplified and cloned into pKLV-U6gRNA5(BbsI)-PGKhyg2ABFP by Gibson assembly[52].

Pooled CRISPR-Cas9 screening

The lentiviral sgRNA library was produced using HEK293T cells as described above. A total of ~120 million cells were transduced with the lentiviral library at a low MOI (<0.3) to ensure that >85% of cells had a single sgRNA integration, resulting in at least 1000X sgRNA representation. Cells were washed to remove the lentivirus the following day and hygromycin selection of the transduced population was applied for a total of 5 days. Doxycycline (1μg/ml) was added to the cells to induce Cas9 expression and start the assay. Cells were propagated on doxycycline for at least 14 population doublings. At least 10 million cells were harvested at the start and end of the assay. Genomic DNA was extracted using the QIAamp DNA mini kit (Qiagen 51304) and amplified for the region containing the sgRNAs. Samples were then purified using Agencourt AMPure XP (Beckman-Coulter A63880) beads to remove primer contamination as per manufacturer’s protocol. Purified samples were

quantified with the Qubit dsDNA HS assay kit (Thermo) and pooled in equimolar concentrations prior to Illumina sequencing on a HiSeq4000 instrument. Sequencing results were aligned using Bowtie and normalised to one million reads. Constructs with normalised counts less than 20 on day 0 were removed from downstream analyses. Relative sgRNA abundances were calculated by dividing normalised sgRNA read counts at the end of the assay with those at the start (day 0). Relative sgRNA abundances were then used to calculate the gene sensitivity score as follows: the mean relative sgRNA abundance of the three most depleted sgRNAs for each gene was calculated and log2 transformed to generate the gene sensitivity score.

CRISPRi library and screening

The pooled libraries contained sgRNA pairs targeting regions of interest along with positive and negative controls using a tandem design previously shown to effectively inhibit enhancer function[16,27]. sgRNAs were designed using the Broad institute sgRNA design tool (https://portals.broadinstitute.org/gpp/public/analysis-tools/sgrna-design). Oligos were ordered from Twist biosciences, amplified and cloned into pKLV2-U6gRNA5(BbsI)-PGKpuro2ABFP using BbsI sites (New England Biolabs). Screening was carried out essentially as described above in the CRISPR-Cas9 screening protocol with puromycin used as a selection marker. For in vivo screening cells were injected subcutaneously into the flanks of NSG mice. Tumours that had grown to approximately 8mm^3 were harvested and homogenized using the Precellys (Bertin instruments). Genomic DNA was extracted using the QIAamp DNA mini kit (Qiagen 51304) and amplified for the region containing the sgRNA1 (unique for each sgRNA pair in the library). Libraries were prepared and sequenced as described in

the CRISPR-Cas9 screening methods. The log2 of the mean of the two most depleted sgRNA pairs for each region of interest was calculated to generate an enhancer sensitivity score. For the in vivo screen, empirical P-values were calculated for each target region by 10,000 permutations of the normalised sgRNA depletion scores (normalised counts in tumour/normalised counts in plasmid) for each tumour.

Analysis of the Achilles dataset

Dependency scores (CERES scores) from genome-wide CRISPR-Cas9 data for 788 cancer cell lines from the Achilles project were downloaded from https://portals.broadinstitute.org/achilles.

Animal studies

All animal experiments were performed in accordance with protocols approved by the Home Office (UK) and the University of Cambridge Animal Welfare and Ethical Review Body (PPL 70/7990 and PFCB122AA). For subcutaneous tumour growth assays, 1-5 x 10^5 cells in 100µL of 1:1 PBS/Matrigel Matrix (BD) solution were injected into each flank of 5-7-week-old athymic nude female mice (Charles River Laboratories 490 (Homozygous)) or for the in vivo CRISPRi screen into 5-7-week-old male NSG mice (Charles River Laboratories, strain: NOD.Cg-Prkdcscid Il2rgtm1WjI/SzJ). Tumour growth was followed by IVIS bioluminescence imaging (Perkin Elmer) and caliper measurement. Tumour volume (V) was calculated using the equation V= (length x width^2) x 0.5. The maximum permitted endpoint of a mean diameter of 12 mm for a superficial tumour was not exceeded in any of the experiments. The housing conditions were as follows: 12/12h dark/light cycle, humidity 45-65%, temperature 20-24°C.

In vitro proliferation assays

For competition assays, control and targeted cells, which carried different fluorescent markers (BFP^+/$mCherry^+$/GFP), were mixed and plated onto multi-well plates in triplicates. The percentage of each cell population was analyzed at T=0 and at multiple time points throughout the assays by flow cytometry on LSR Fortessa (BD Biosciences). The following gating approach was used: FSC-A, FSC-W, SSC-A to select for live and single cells, and then mCherry (561nm/610nm), BFP (383nm/445nm) or GFP (488nm/510nm) channels for discriminating between the cell populations (Supplementary Figure 4).

mRNA and protein detection

Total RNA was extracted from cells using the RNAzol®RT reagent (Sigma) or RNeasy Mini kit (Qiagen) as per the manufacturer's protocol and 500ng was used to generate cDNA using the High-Capacity cDNA Reverse Transcription Kit (Thermo). qPCR was performed using Taqman reagents (Thermo) on the StepOnePlus™ Real Time PCR instrument (Thermo). The following TaqMan gene expression assays were used: *HNF1B* (Hs01001602_m1), *PAX8* (Hs00247586_m1), *TBP* (Hs00427620_m1), *MYC* (Hs00153408_m1), *VHL* (Hs03046964_s1) and *CCND1* (Hs00765553_m1). *TBP* was used as a housekeeping control. Relative gene expression was calculated using the $2^{-\Delta\Delta Ct}$ method. Total protein was extracted from cell pellets using RIPA buffer (Sigma-Aldrich) containing protease and phosphatase inhibitor cocktail (Sigma-Aldrich) according to the manufacturer's protocol. Proteins were separated by SDS-PAGE, transferred onto PVDF membrane (Millipore) and blotted with PAX8 (Santa Cruz Biotech, sc-81353 1:250), HNF1B (Human Protein

Atlas, HPA002083 1:5000), MYC (Abcam, ab32072, 1:1000), HIF2A (Novus Biologicals, NB100-122, 1:1000), VHL (BD Pharmingen, 565183, 1:1000), CCND1 (Abcam, ab134175, 1:1000), HIF1A (R&D systems, MAB1536, 1:500), and B-actin (Sigma-Aldrich, A1978, 1:20000) antibodies. Secondary antibodies were polyclonal goat anti-mouse IgG/HRP (Dako, P0447, 1:10000) and polyclonal goat anti-rabbit IgG/HRP conjugated (Dako, P0448, 1:5000). Protein expression was quantified using Image J and normalised to B-actin control. For gel source data, see Supplementary Figure 1 and 2.

Immunohistochemistry

Tumour xenografts were collected and fixed overnight in 4% paraformaldehyde, washed, embedded in paraffin, and sectioned. Human Vimentin (Cell Signaling Technology; cat. 5741, 1:100) and HIF2A (Santa Cruz sc-46691,1:200) staining was performed in a Bond-Max instrument (Leica) using Bond Polymer Refine Detection reagents (Leica) according to the manufacturer's protocol (IHC Protocol F).

Tissue microarray

A tissue microarray of human ccRCCs representing the full range of stages of disease and healthy adult kidney, created as previously reported[53], was used for PAX8 and HNF1B immunohistochemistry. Ethical approval to use these archived tissues was granted by the Lothian Regional Ethics Committee (08/S1101/41 and 10/S1402/33). Patient age at sampling was 28-91 years with 40% females and 60% males. Immunohistochemistry was performed on 3μm sections cut from formalin-fixed paraffin embedded tissue blocks, using a fully automated system (BOND III IHC and ISH stainer from Leica). The PAX8 primary antibody clone MRQ-50 (363M-

16, Cell Marque) and the HNF1B primary antibody (Human Protein Atlas, HPA002083) were used at a dilution of 1:100 after heat-induced epitope retrieval at pH 9.0 (BOND epitope retrieval solution 2, Leica) for 30 minutes. The Primary antibody binding to tissue sections was visualized using BOND Polymer Refine Detection (Leica). PAX8 and HNF1B staining were scored according to percentage positive nuclear staining using the following grades: 0 (no staining), 1 (<10% positive cells), 2 (10-50% positive cells) and 3 (>50% positive cells).

Co-immunoprecipitation

Nuclear proteins from C-M1A$^{HIF2A-/-}$ cells with HIF2A reintroduction were prepared using the NE-PER Nuclear and cytoplasmic extraction kit (Pierce 78833) according to manufacturer's protocol. For IP, protein A/G magnetic beads (Thermo, 26162) were washed in RIPA buffer (Sigma-Aldrich) three times then incubated with fresh pre-cleared nuclear lysate and antibodies PAX8 (ProteinTech 10336-1-AP), HIF2A (Abcam, ab199) or rabbit polyclonal IgG (Abcam, ab27478) at 4°C while rotating for 2 hours. The protein bound-beads were then washed in cold lysis buffer three times, eluted in 2X SDS loading buffer, boiled for 5 minutes and analysed by SDS-PAGE using the following antibodies: PAX8 (Santa Cruz Biotech, sc-81353 1:250), HIF2A (Novus Biologicals, NB100-122, 1:1000), HIF1B (Santa Cruz Biotech, H-10, sc-55526, 1:200). Secondary antibodies used were anti-mouse IgG/HRP for IP (Abcam, ab131368, 1:5000) and VeriBlot for IP detection (HRP) (Abcam, ab131366, 1:5000).

Chromatin immunoprecipitation

Approximately 30mg of tumour tissue was homogenized in the Precellys (Bertin instruments) for 30sec and crosslinked with 1% formaldehyde-supplemented media

for 10 minutes. The reaction was quenched with 0.125M glycine for 5 minutes and followed by PBS washes twice. The cells were either pelleted and stored at -80°C or subjected to immunoprecipitation. For IP, the protein A/G magnetic beads (Thermo, 26162) were first equilibrated by washing the beads with 0.5% BSA in PBS three times. The beads were then incubated with antibodies in 0.5% BSA in PBS at 4°C while rotating for a minimum of 4 hours. The crosslinked cells were resuspended and dounced in lysis buffer (20 mM Tris-HCl pH8.0, 150 mM NaCl, 2mM EDTA pH 8.0, 0.1% SDS and 1% Triton X-100), followed by sonication in the Bioruptor (Diagenode) for 14 cycles, 30” on / 30” off. The lysates were spun down at 4°C for 20 minutes at 14,000 rpm. The supernatants were added onto the antibody-conjugated magnetic beads and incubated overnight at 4°C while rotating. On the following day, the beads were washed three times with low salt buffer (50 mM HEPES pH 7.5, 140 mM NaCl, 1% Triton) and once with high salt buffer (50 mM HEPES pH 7.5, 500 mM NaCl, 1% Triton). The DNA bound to the antibody-conjugated beads was eluted with elution buffer (50 mM $NaHCO_3$, 1% SDS) and de-crosslinked by shaking at 1,000 rpm for 3 hours at 65°C. De-crosslinked DNA was purified using the QuickClean II PCR Extraction Kit (Genescript L00419-100) according to the manufacturer’s recommendations. For in vitro cell lines, ChIP was performed using the iDeal ChIP kit for transcription factors (Diagenode) according to the manufacturer’s protocol. The following antibodies were used for all ChIP assays: PAX8 (ProteinTech 10336-1-AP), HNF1B (Human Protein Atlas, HPA002083), HIF2A (Novus Biologicals NB100-122) and rabbit polyclonal IgG (Abcam, ab27478). For allele specific qPCRs, genotyping Taqman assays were used for SNPs rs7948643 and rs7177 (Thermo). All other qPCR primer sequences are available in Supplementary Table 10.

ChIP-seq library preparation

Purified ChIP DNA was subjected to Illumina sequencing. Sequencing libraries were prepared using the KAPA Hyper Prep Kit (KR0961) according to the manufacturer's recommendations. Adapter-ligated libraries were size-selected using Agencourt AmPure XP beads (Beckman Coulter A63880) to obtain fragments of 150-350bp. Size-selected fragments were amplified for 15 cycles using the KAPA HiFi HotStart Ready mix and the amplified libraries were pooled in equimolar concentration for Illumina sequencing on a HiSeq4000 instrument.

RNA-seq

Total RNA was extracted from sub-confluent cells in four replicates using the RNeasy Mini Kit (Qiagen 74104) according to the manufacturer's protocols. RNA concentration and quality were assessed with the Agilent RNA Nano 6000 kit (Agilent 5067-1511) on an Agilent Bioanalyzer 2100 instrument. RNA-Seq libraries were prepared using the SENSE/CORALL mRNA-Seq Library Prep Kits (Lexogen) following the manufacturer's recommendations with 1μg of total RNA as the starting material. The size and quality of the final library products were assessed using the Agilent High Sensitivity DNA Kit (Agilent 5067-4626). Library concentration was determined using the KAPA Library Quantification Kit (KR0405). Libraries were pooled in equimolar concentrations and subjected to Illumina sequencing on a HiSeq4000 instrument. Cell line RNA-seq sequencing reads were mapped to hg38 using RSEM[54] and bowtie2. For xenograft RNA-seq reads mapping to mouse transcriptome (mm10) were first filtered out using bowtie2, after which the remaining reads were mapped to hg38. Differentially expressed genes were identified using DESeq2 (ref. [55]). HIF2A binding sites were identified within the 500kb regions flanking the transcription start sites of the 205 consistent (i.e. downregulated at 32h

and 72h after dox withdrawal) in vivo HIF2A target genes, and enrichment of HIF2A binding sites within these genomic loci was evaluated by randomly picking 1,000 sets of 205 expressed genes (empirical P-value < 0.001). PAX8 and HNF1B signatures were defined as genes downregulated (logFC<(-0.58), adjusted P-value <0.05) in both of the cell lines, 786-M1A and OS-LM1. Gene set enrichment analysis was performed using the R packages ClusterProfiler[56] and Molecular Signature Database (MSigDB) Hallmarks gene set (version 7.1.1).

Electrophoretic mobility shift assay

Nuclear proteins from MDA-MB-231 cells transduced with either an empty vector or a PAX8-expressing vector were prepared using the NE-PER Nuclear and cytoplasmic extraction kit (Pierce 78833). Electrophoretic mobility shift assays were performed with 6% polyacrylamide gel using LightShift Chemiluminescent EMSA kit as per manufacturer's protocol (Pierce 20148). Double-stranded DNA probes with biotin at one end of the strands were prepared using an annealing buffer (10 mM Tris/HCl, 50 mM NaCl, 1mM EDTA, pH 8.0) and heating to 95°C for 5 min and subsequent cooling at 1°C/min to 20°C in a thermocycler. Protein samples and labelled DNA were incubated for 45 min at room temperature in binding buffer. Gels were first pre-run using at 100 V for 60 min, and then samples were loaded and gels were run for 60 min at 180 V in 4°C and transferred to a nylon membrane (Pierce 77016) for 60 min at 380 mA. Oligos used are listed in Supplementary Table 10. For gel source data, see Supplementary Figure 3.

ATAC-seq

Cells were treated with 1µg/ml doxycycline for 10 days prior to harvest. Cells were seeded in standard culture conditions to allow correct attachment and ensure 75% confluency at harvesting day. Cells were trypsinized, counted and 50,000 cells were subjected to the ATAC-seq protocol directly as previously described[57]. Libraries were prepared using the Illumina Nextera DNA library preparation kit (FC-121-1030) and purified using the minElute PCR purification kit (Qiagen 28004). Following purification, library fragments were amplified using NEBNext PCR master mix and custom Nextera PCR primers. Libraries were amplified for a total of 8-12 cycles and cleaned-up using the Agencourt AMPureXP reagent (A63880) according to manufacturer's recommendations and pooled in equimolar concentrations prior to sequencing.

Reporter Assays

Enhancer activity was determined by luciferase reporter assays. The region comprising the E11:69419 peak (approximately 350 bp) was cloned into the STARR-seq luciferase validation vector_ORI_empty (Addgene plasmid # 99297) containing a Firefly luciferase using the following primers: 5'-ATCGGGTACCTTAGACCAAAGCGACAGTGT-3' and 5'-ATCGCTCGAGGCTGCAGAGGAACATCGTTT-3'. Genomic DNA from 786-O cells was used as template. ccRCC cells were co-transfected with 3.6 µg of enhancer reporter plasmid and 400 ng of pRL-TK Renilla luciferase vector (Promega E2241) as a normalisation control using an Amaxa Nucleofector (programme T-20, reagent V, Lonza). After 48 hours of incubation, luciferase activity was measured using the Dual-Glo Luciferase assay (Promega, E2920). Mutation of the HIF2A and PAX8 binding sites were generated using the QuickChange II site-directed mutagenesis kit

(Agilent) with the following primers: 5′-CAGAGCTGGTTCTGCAAATACCTCCGCTCCACAG-3’ and 5’-CTGTGGAGCGGAGGTATTTGCAGAACCAGCTCTG-3 for HIF2A-1, 5’-CTGAGGTGCTCAGGGGTCGGCAATCAAAAAAACTGGGCAGCCGTACGTGTCC-3’ and 5’-GGACACGTACGGCTGCCCAGTTTTTTTGATTGCCGACCCCTGAGCACCTCAG-3’ for PAX8-1, 5’- GATCACTGGGCAGCCGTAAAAGTCCCAGGCAGCGG-3’ and 5’-CCGCTGCCTGGGACTTTTACGGCTGCCCAGTGATC-3’ for HIF2A-2, 5’-CCGGCAGTGCCAGCTCCCACACAATAAAACTATGACAACCTCAGGTCCTTGATG-3’ and 5’-CATCAAGGACCTGAGGTTGTCATAGTTTTATTGTGTGGGAGCTGGCACTGCCGG-3’ for PAX8-2, 5’-CTGAGGTGCTCAGGGGTCGGCGCTCAGGATCACCGGGCAGCCGTACGTGTCC-3’ and 5’-GGACACGTACGGCTGCCCGGTGATCCTGAGCGCCGACCCCTGAGCACCTCA-3’ for SNP T>C.

Organoid culture

Kidney organoid medium was prepared from Advanced DMEM/F12 (Gibco) supplemented with 1.5% B27 supplement (Gibco), 40% Wnt3A conditioned medium, 10% RSPO-conditioned medium, EGF (50ng/ml, Proteintech), FGF-10 (100ng/ml, Proteintech), N-acetylsysteine (1.25mM, Sigma), Rho-kinase inhibitor Y-27632 (10μM, APEBIO), Noggin (100ng/ml Proteintech), A8301 (5μM,TOCRIS) and Primocin (100ng/ml, InvivoGen).

Normal human kidney tissue was sampled, with informed consent, from a 75-year-old male by a consultant uropathologist within 2 hours of nephrectomy under an ethical approval by the East of England - Cambridge Central Research Ethics Committee (19/EE/0161). The tissue was transported on ice in cold HBSS, dissected in PBS supplemented with penicillin (100U/mL)/streptomycin (100μg/mL) and further split into advanced DMEM/F12 (Gibco). Kidney tissue pieces were minced, washed in wash medium (advanced DMEM/F12 supplemented with 1X Glutamax, penicillin (100U/mL), streptomycin (100μg/mL) and 10mM Hepes) and resuspended in kidney organoid medium containing collagenase A (1mg/mL, Sigma) for 15 minutes at 37°C with shaking. The cells were washed, pelleted by centrifugation (5 minutes, 300 rcf, 4°C), resuspended in wash medium, passed through a 70μm strainer, pelleted by centrifugation (5 minutes, 300 rcf, 4°C) and resuspended in 100μl wash medium. Single cells were seeded in 70% growth factor-reduced BME (R&D systems) and cast into 20μl droplets in a 12-well plate. After polymerization of the BME (30 minutes, 37°C), 1mL kidney organoid medium was added to each well. For passage, organoids were dissociated using 1mL TrypLE (Gibco) containing 10μM Y-27632 per well (InvivoGen). TrypLE dissociation was stopped by adding 10mL advanced DMEM/F12 and centrifuged at 300 rcf for 5 minutes, cells were reseeded in fresh 70% BME and topped with kidney organoid medium.

Lentiviral transduction for organoids

Lentiviral supernatant was collected and mixed with LentiX concentrator (TaKaRa) in a ratio of 3:1. The mixture was incubated for 30 minutes at 4°C, and centrifuged at 4°C (1500 rcf, 1 hour). Virus pellets were resuspended in kidney organoid medium with polybrene (5μg/ml). Organoids were dissociated into single cells and organoid

pellets were resuspended in concentrated virus in 15 ml falcon tubes and incubated for 4 hours, then the falcon tubes were centrifuged at 600 rcf at 32°C for 1 hour before reseeding organoids in fresh 70% BME.

Long read sequencing and data analysis

High Molecular Weight (HMW) genomic DNA was extracted from the RCC-JF cells using phenol-chloroform extraction. 30μl of HMW genomic DNA was then put onto a SageELF 0.75% Agarose Cassette and loaded onto a sageELF instrument using a running time of 7 hours, 10-40kb run mode. All 12 elution wells were added together (5-60kb). The sample was 1X SPRI cleaned and eluted in 48μl of PCR grade water. The Genomic DNA by Ligation protocol SQK-LSK109 was followed with the following changes. The DNA end-repair reaction was extended to 20 minutes. The adaptor ligation reaction was extended to 20 minutes. SFB was used to wash the final complex. Final adapted library was loaded at 7-10 fmol on the PromethION using a single cell and run for 72 hours following manufacturer's recommendations.

Variant calling was performed on the aligned reads using *longshot* (v. 0.4.1)[58], with default parameters. The reference file was downloaded from UCSC (https://hgdownload.soe.ucsc.edu/goldenPath/hg38/chromosomes/). Once the variants of interest were identified, the alignments were queried read by read using the python package *pysam* (v. 0.16) (https://github.com/pysam-developers/pysam), a wrapper around the htslib[59] and samtools[60] packages, to phase the reads by risk and protective alleles. Only reads with no sequencing error in the variants of interest were selected for visualisation.

Fluorescent in situ hybridisation

Cell pellets were resuspended in 0.075M KCl, incubated for 15 minutes at 37°C, spun down (1800 rpm, 7 minutes) and resuspended in 5% acetic acid. Cells were pelleted by centrifugation (1800 rpm, 7 minutes), resuspended in ice-cold Carnoy's fixative (3:1 Methanol:Acetic Acid, prepared fresh) and stored at -20°C for at least 30 minutes. Fixed cell suspension was dropped onto a clean microscope slide and fluorescent in situ hybridisation analyses were performed using the Vysis LSI MYC Dual Colour, Break apart rearrangement probe (Abbott Molecular).

scRNA-seq data analysis

Gene expression count matrix of fetal kidney was downloaded[61] and cells labelled as CD45 negative fetal samples (4834STDY7002876, 4834STDY7002881, 4834STDY7002886) were processed. Cells that had greater than 20% mitochondrial gene expression or expressed fewer than 1,500 distinct transcripts were removed. Filtered count matrices were normalised using the SCTransform[62] function. 3,000 integration anchors were identified across samples using a function called FindIntegrationAnchors from the Seurat package[63,64]. The identified anchors were then used for batch correction (Seurat function IntegrateData, normalisation.method="SCT"). The first 30 principal components were used to calculate UMAPs and to perform clustering using FindNeighbors and FindClusters (resolution=0.3) functions. Cell types were assigned by manually checking the expression of previously reported marker genes[65–67]. A cluster that showed less than 15 marker genes (FindMarkers, logfc.threshold=0.25) and CD45+ immune cell cluster were removed. The R packages Tidyverse, Rcolorbrewer and

ComplexHeatmap were used to make UMAP and heatmap plots. In the trajectory analysis, lineage inference was performed applying the slingshot function from the Slingshot package and using clusters identified by Seurat and UMAP as the dimensionality reduction[68]. The nephron progenitor cell cluster was set as the origin of the trajectory. Expression of *PAX8* and *HNF1B* along the renal epithelial cell lineage was modelled using the tradeSeq package[69]. R package ggbeeswarm was used for making the plots combining pseudotime with gene expression. PAX8 and HNF1B signatures were mapped to scRNA-seq data using the AddModuleScore function (Seurat package).

ATAC-seq data analysis

Adapter contaminations identified by FastQC (version 0.11.9) were removed, low quality bases were trimmed from read ends (quality < 20) and reads shorter than 10 bases were discarded using cutadapt (version 2.10)[70,71]. Reads were then mapped to human reference genome (hg38) using BWA (version 0.7.17)[72]. The output SAM files were converted to bigwig format using Samtools (version 1.9) (SAM -> BAM) and deepTools (version 3.5.0) (BAM -> bigwig). Low quality reads (mapping quality < 20), reads mapping to chromosomes other than chromosomes 1 to 22, X and Y and blacklisted regions were further removed[72–74]. Reads were corrected for the Tn5 offset (+ve strand: +4bp, -ve strand: -5bp). Peak calling was performed using MACS2 (version 2.2.7.1) with parameters "-f BAM –bdg -g 2913022398 –nomodel –nolambda –shift -100 –extsize 200" [75].

ATAC-seq peaks were combined by first extending them to a fixed 500bp window flanking the peak summit. All peaks across the different experimental conditions and

replicates were then ranked based on their q-value, and moving down from the most significant peak, each less significant but overlapping peak was removed, resulting in a consensus peak set in which the precise position of a peak was defined by the most significant peak called at that locus. Reads were extended to a modal length of 250bp and count tables for unique reads were generated for the consensus peak set using RSamtools. Peaks with $-\log_{10}(q)<20$ were filtered out and differentially accessible peaks (FC+/-1.25 and padj<0.05) were called using DESeq2 (ref. [55]). Enriched de novo and known motifs within the regions of increased or decreased accessibility upon PAX8 and HNF1B depletion were identified using homer[76], with a non-changed peak set (FC less than +/-1.25 and padj>0.3) as a reference. For the TCGA cohort of ATAC-seq samples[23], count tables and normalised signal tables for the entire cohort were downloaded from https://gdc.cancer.gov/about-data/publications/ATACseq-AWG, duplicate samples were merged, and cancer type-specific peaks were identified using DESeq2 (ref. [55]) with each cancer type individually being compared to all other cancer types. Overlap with PAX8 and HNF1B-dependent peaks was identified using bedops[77]. The significance of overlap was tested by Fisher's exact test in R. The modules ame and fimo from the MEME suite[78] were used for motif scanning and enrichment analysis, respectively. For allele-specific chromatin accessibility analysis non-unique reads were removed using *samtools* from bam files and the risk and protective allele fractions were calculated as the average of the allele fractions of rs7948643 and rs7939721. Heatmaps were generated using normalised coverage of peaks (+/-1kb) with the deepTools package (version 3.5.0). Normalised signal for DNase I hypersensitivity across human tissues was obtained from ref. [29].

ChIP-seq data analysis

Adapter contaminations identified by FastQC (version 0.11.7) were removed, low quality bases were trimmed from read ends (quality < 20) and reads shorter than 10 bases were discarded using Cutadapt (version 1.10.0). Reads were first mapped to the mouse reference genome (mm10) with Bowtie2 (version 2.3.4.3)[79] and only the reads which did not map to mm10 were used for further analysis. Reads which did not map to mm10 were mapped to hg38 with 'sponge' regions using Bowtie2 (ref. [80]). The output SAM files were converted to bigwig format using Samtools (version 1.2). Low quality reads (mapping quality < 20), reads mapping to chromosomes other than chr1 to 22, X and Y and blacklisted regions were then removed. Peak calling was performed with MACS2 (version 2.2.7.1).

High-confidence peak sets for each condition were identified using peak regions that were present in at least two replicates with bedops[77]. TF binding regions were identified by extending summits within the high-confidence peak regions by -50bp and +49bp and merging the overlapping regions using the R packages dplyr and bedtools[81]. These regions were then used to calculate the number of overlapping regions between two TFs. Heatmaps were generated using normalised coverage of peaks (+/- 2kb) using the deepTools package (version 3.5.0).

RIME

For the proteomic analysis, the samples were prepared as has been previously described[21]. The following antibodies were used: PAX8 (ProteinTech 10336-1-AP), HIF2A (Novus Biologicals NB100-122) and rabbit polyclonal IgG (Abcam, ab27478). Briefly, bead-bound proteins were digested with the addition of 10µl trypsin (15ng/µl, Pierce) in 100mM ammonium bicarbonate (AMBIC), followed by overnight incubation

at 37°C. The next day, a second digestion step was performed by adding again trypsin solution for 4h at 37°C. The peptides were acidified with the addition of 5% formic acid and purified using the Ultra-Micro C18 Spin Columns (Harvard Apparatus) according to the manufacturer's instructions. After the purification the samples were dried with speedvac concentrator and reconstituted in 20μl 0.1% formic acid prior to mass spectrometry analysis. For the analysis of the peptides, the Dionex Ultimate 3000 UHPLC system coupled with the Q-Exactive mass spectrometer (Thermo Scientific) was used. The collected HCD tandem mass spectra were processed with the SequestHT search engine on the Proteome Discoverer 1.4 software. All spectra were searched against a UniProtKB/Swiss-Prot fasta file containing 20,394 reviewed human entries and the node for SequestHT included the following parameters: Precursor Mass Tolerance 20ppm, Fragment Mass Tolerance 0.02Da, Dynamic Modifications were Oxidation of Methionine (M) (+15.995 Da) and Deamidation of Asparagine (N) and Glutamine (Q) (+0.984 Da). Proteins detected in IgG control RIME were excluded from further analysis whereas proteins detected at least in three out of four replicates for HIF2A and PAX8 RIME were included. Nuclear proteins were identified using the Gene Ontology resource[82,83], and the highest confidence experimental and database-derived physical connections between PAX8 and HIF2A shared hits were identified using String 11.0, followed by MCL clustering[84].

Genomewide association study (GWAS) data

RCC subtype-specific SNP information for the haplotype containing the RCC risk marker rs7105934 (chr11:69,419,819-69,437,482 in hg38) and rs7948643 was extracted for ccRCC and papillary RCC from the summary data of a previously

published RCC GWAS meta-analysis[5]. The combined GWAS data set evaluating subtypes comprised a total of five previously published scans with data on tumour histology: two from the National Cancer Institute (NCI-1, 1,311 cases and 3,424 controls[4]; NCI-2, 2,417 cases and 5,071 controls[5]), two from the International Agency for Research on Cancer (IARC-1: 2,438 cases and 5,071 controls[4]; IARC-2: 2,781 cases and 2,940 controls[5]) and one from the University of Texas MD Anderson Cancer Center (MDA: 893 cases and 556 controls[85]). The sixth scan in the original GWAS did not include subtype data[86]. In the original meta-analysis, imputation was performed separately for each scan data set with SNPs of minor allele frequency ≥0.01 (≥0.05 for the IARC data set) using 1000 Genomes Project data (phase 1 release 3). Minimac version 3 was used for imputation of the IARC data set (IARC-1 and IARC-2 combined) whereas IMPUTE2 version 2.2.2 was used for the other data sets[87,88]. Imputed SNPs with $r^2 \geq 0.3$ for both IMPUTE2 and Minimac (n=7,437,091) were retained for data analysis. For the present study, RCC subtype information was identified for a total of 5,648 ccRCCs and 15,010 corresponding controls and 563 papillary RCCs and 14,840 corresponding controls across the complete RCC meta-analysis data set[5]. Association analysis for ccRCC and papillary RCC was conducted separately for each data set assuming log-additive (trend) SNP effects using R version 3.2.3 for the IARC data set and SNPTEST version 2.2 for the other data sets. The ccRCC model covariates were as follows: sex and study for NCI-1 (no statistically significant eigenvectors present in null model); sex and four significant eigenvectors for NCI-2; age, sex and two significant eigenvectors for MDA; and sex, study, and 19 significant eigenvectors for IARC-1 and IARC-2. Eigenvectors were considered significant if $P<0.05$ from the Tracy–Widom statistics. The papillary RCC model covariates were as follows: sex, study and two significant

eigenvectors for NCI-1; sex and three significant eigenvectors for NCI-2; age, sex and one significant eigenvector for MDA; and sex, study, and significant eigenvectors for IARC-1 and IARC-2. The SNP association results from each data set were combined by meta-analysis using a fixed-effects model. Heterogeneity in genetic effects across data sets was assessed using the I^2 and Cochran's Q statistics. The regional association plots were generated using LDassoc[89] for SNPs with a minor allele frequency ≥0.01 for ccRCC and ≥0.05 for papillary RCC. The regulatory potential was estimated by RegulomeDB[90].

Statistical analysis

Statistical analyses were performed in R, Excel or GraphPad Prism. P-values lower than 0.05 were considered statistically significant. No randomization or blinding was used. No formal approach for sample size calculation was used. For normalised data non-parametric tests were used. For competition assays, reporter assays, qRT-PCR analysis and tumour growth data Kruskal-Wallis test was used, with Dunn's multiple comparison test applied for data sets with more than one pairwise comparison. For qRT-PCR and ChIP qPCR, individual data points represent the mean of 2-3 technical replicates. For correlation analyses, Pearson correlation coefficient was calculated. Logrank test was used for categorical survival data.

43. Wang, T., Wei, J. J., Sabatini, D. M. & Lander, E. S. Genetic Screens in Human Cells Using the CRISPR-Cas9 System. *Science* **343**, 80–84 (2014).

44. Sanjana, N. E., Shalem, O. & Zhang, F. Improved vectors and genome-wide libraries for CRISPR screening. *Nat. Methods* **11**, 783–784 (2014).

45. Gilbert, L. A. *et al.* Genome-Scale CRISPR-Mediated Control of Gene Repression and Activation. *Cell* **159**, 647–661 (2014).

46. Koike-Yusa, H., Li, Y., Tan, E. P., Velasco-Herrera, M. D. C. & Yusa, K. Genome-wide recessive genetic screening in mammalian cells with a lentiviral CRISPR-guide RNA library. *Nat. Biotechnol.* **32**, 267–273 (2014).

47. Tzelepis, K. *et al.* A CRISPR Dropout Screen Identifies Genetic Vulnerabilities and Therapeutic Targets in Acute Myeloid Leukemia. *Cell Rep.* **17**, 1193–1205 (2016).

48. Fellmann, C. *et al.* An optimized microRNA backbone for effective single-copy RNAi. *Cell Rep.* **5**, 1704–1713 (2013).

49. Muerdter, F. *et al.* Resolving systematic errors in widely used enhancer activity assays in human cells. *Nat. Methods* **15**, 141–149 (2018).

50. Sauert, K. *et al.* Heat-Shock Mediated Overexpression of HNF1β Mutations Has Differential Effects on Gene Expression in the Xenopus Pronephric Kidney. *PLoS One* **7**, e33522 (2012).

51. Vaquerizas, J. M., Kummerfeld, S. K., Teichmann, S. A. & Luscombe, N. M. A census of human transcription factors: function, expression and evolution. *Nat. Rev. Genet.* **10**, 252–263 (2009).

52. Gibson, D. G. *et al.* Enzymatic assembly of DNA molecules up to several hundred kilobases. *Nat. Methods* **6**, 343–345 (2009).

53. Laird, A. *et al.* Differential Expression of Prognostic Proteomic Markers in Primary Tumour, Venous Tumour Thrombus and Metastatic Renal Cell Cancer Tissue and Correlation with Patient Outcome. *PLoS One* **8**, e60483 (2013).

54. Li, B. & Dewey, C. N. RSEM: accurate transcript quantification from RNA-Seq data with or without a reference genome. *BMC Bioinformatics* **12**, 323 (2011).

55. Love, M. I., Huber, W. & Anders, S. Moderated estimation of fold change and dispersion for RNA-seq data with DESeq2. *Genome Biol.* **15**, 550 (2014).

56. Yu, G., Wang, L.-G., Han, Y. & He, Q.-Y. clusterProfiler: an R Package for Comparing Biological Themes Among Gene Clusters. *Omi. A J. Integr. Biol.* **16**, 284–287 (2012).

57. Buenrostro, J. D., Giresi, P. G., Zaba, L. C., Chang, H. Y. & Greenleaf, W. J. Transposition of native chromatin for fast and sensitive epigenomic profiling of open chromatin, DNA-binding proteins and nucleosome position. *Nat. Methods* **10**, 1213–1218 (2013).

58. Edge, P. & Bansal, V. Longshot enables accurate variant calling in diploid genomes from single-molecule long read sequencing. *Nat. Commun.* **10**, 4660 (2019).

59. Bonfield, J. K. *et al.* HTSlib: C library for reading/writing high-throughput sequencing data. *Gigascience* **10**, (2021).

60. Danecek, P. *et al.* Twelve years of SAMtools and BCFtools. *Gigascience* **10**,

(2021).

61. Young, M. D. *et al.* Single-cell transcriptomes from human kidneys reveal the cellular identity of renal tumors. *Science* **361**, 594–599 (2018).

62. Hafemeister, C. & Satija, R. Normalization and variance stabilization of single-cell RNA-seq data using regularized negative binomial regression. *Genome Biol.* **20**, 296 (2019).

63. Stuart, T. *et al.* Comprehensive Integration of Single-Cell Data. *Cell* **177**, 1888-1902.e21 (2019).

64. Butler, A., Hoffman, P., Smibert, P., Papalexi, E. & Satija, R. Integrating single-cell transcriptomic data across different conditions, technologies, and species. *Nat. Biotechnol.* **36**, 411–420 (2018).

65. Wang, P. *et al.* Dissecting the Global Dynamic Molecular Profiles of Human Fetal Kidney Development by Single-Cell RNA Sequencing. *Cell Rep.* **24**, 3554–3567.e3 (2018).

66. Lindström, N. O. *et al.* Progressive Recruitment of Mesenchymal Progenitors Reveals a Time-Dependent Process of Cell Fate Acquisition in Mouse and Human Nephrogenesis. *Dev. Cell* **45**, 651–660.e4 (2018).

67. Hochane, M. *et al.* Single-cell transcriptomics reveals gene expression dynamics of human fetal kidney development. *PLoS Biol.* **17**, e3000152 (2019).

68. Street, K. *et al.* Slingshot: cell lineage and pseudotime inference for single-cell transcriptomics. *BMC Genomics* **19**, 477 (2018).

69. den Berge, K. *et al.* Trajectory-based differential expression analysis for single-cell sequencing data. *Nat. Commun.* **11**, 1201 (2020).

70. Martin, M. Cutadapt removes adapter sequences from high-throughput sequencing reads. *EMBnet.journal* **17**, 10–12 (2011).

71. Andrews, S. & Others. FastQC: a quality control tool for high throughput sequence data. (2010).

72. Li, H. Aligning sequence reads, clone sequences and assembly contigs with BWA-MEM. (2013).

73. Ramírez, F. *et al.* deepTools2: a next generation web server for deep-sequencing data analysis. *Nucleic Acids Res.* **44**, W160-5 (2016).

74. Amemiya, H. M., Kundaje, A. & Boyle, A. P. The ENCODE Blacklist: Identification of Problematic Regions of the Genome. *Sci. Rep.* **9**, 9354 (2019).

75. Zhang, Y. *et al.* Model-based analysis of ChIP-Seq (MACS). *Genome Biol.* **9**, R137 (2008).

76. Heinz, S. *et al.* Simple Combinations of Lineage-Determining Transcription Factors Prime cis-Regulatory Elements Required for Macrophage and B Cell Identities. *Mol. Cell* **38**, 576–589 (2010).

77. Neph, S. *et al.* BEDOPS: high-performance genomic feature operations. *Bioinformatics* **28**, 1919–1920 (2012).

78. Bailey, T. L. *et al.* MEME SUITE: tools for motif discovery and searching. *Nucleic Acids Res.* **37**, W202–W208 (2009).

79. Langmead, B. & Salzberg, S. L. Fast gapped-read alignment with Bowtie 2. *Nat. Methods* **9**, 357–359 (2012).

80. Miga, K. H., Eisenhart, C. & Kent, W. J. Utilizing mapping targets of sequences underrepresented in the reference assembly to reduce false positive alignments. *Nucleic Acids Res.* **43**, e133 (2015).

81. Quinlan, A. R. & Hall, I. M. BEDTools: a flexible suite of utilities for comparing genomic features. *Bioinformatics* **26**, 841–842 (2010).

82. Ashburner, M. *et al.* Gene Ontology: tool for the unification of biology. *Nat. Genet.* **25**, 25–29 (2000).

83. Carbon, S. *et al.* The Gene Ontology resource: enriching a GOld mine. *Nucleic Acids Res.* **49**, D325–D334 (2021).

84. Szklarczyk, D. *et al.* STRING v11: protein–protein association networks with increased coverage, supporting functional discovery in genome-wide experimental datasets. *Nucleic Acids Res.* **47**, D607–D613 (2019).

85. Wu, X. *et al.* A genome-wide association study identifies a novel susceptibility locus for renal cell carcinoma on 12p11.23. *Hum. Mol. Genet.* **21**, 456–62

(2012).

86. Henrion, M. *et al.* Common variation at 2q22.3 (ZEB2) influences the risk of renal cancer. *Hum. Mol. Genet.* **22**, 825–31 (2013).

87. Howie, B., Fuchsberger, C., Stephens, M., Marchini, J. & Abecasis, G. R. Fast and accurate genotype imputation in genome-wide association studies through pre-phasing. *Nat. Genet.* **44**, 955–9 (2012).

88. Howie, B. N., Donnelly, P. & Marchini, J. A flexible and accurate genotype imputation method for the next generation of genome-wide association studies. *PLoS Genet.* **5**, e1000529 (2009).

89. Machiela, M. J. & Chanock, S. J. LDassoc: an online tool for interactively exploring genome-wide association study results and prioritizing variants for functional investigation. *Bioinformatics* **34**, 887–889 (2018).

90. Boyle, A. P. *et al.* Annotation of functional variation in personal genomes using RegulomeDB. *Genome Res.* **22**, 1790–7 (2012).
