# Supplementary material for: The renal lineage factor PAX8 controls oncogenic signalling in kidney cancer
Source: Nature. 2022 Jun 8;606(7916):999–1006. doi: 10.1038/s41586-022-04809-8 (PMC9242860; doi:10.1038/s41586-022-04809-8)
Supplement: Supplementary file 2 — Reporting Summary [file 41586_2022_4809_MOESM2_ESM.pdf]

nature research

Corresponding author(s): Sakari Vanharanta

Last updated by author(s): Mar 20, 2022

Reporting Summary

Nature Research wishes to improve the reproducibility of the work that we publish. This form provides structure for consistency and transparency in reporting. For further information on Nature Research policies, see our Editorial Policies and the Editorial Policy Checklist.

Statistics

For all statistical analyses, confirm that the following items are present in the figure legend, table legend, main text, or Methods section.

n/a	Confirmed	
☐	☒	The exact sample size (*n*) for each experimental group/condition, given as a discrete number and unit of measurement
☐	☒	A statement on whether measurements were taken from distinct samples or whether the same sample was measured repeatedly
☐	☒	The statistical test(s) used AND whether they are one- or two-sided<br>*Only common tests should be described solely by name; describe more complex techniques in the Methods section.*
☒	☐	A description of all covariates tested
☐	☒	A description of any assumptions or corrections, such as tests of normality and adjustment for multiple comparisons
☐	☒	A full description of the statistical parameters including central tendency (e.g. means) or other basic estimates (e.g. regression coefficient) AND variation (e.g. standard deviation) or associated estimates of uncertainty (e.g. confidence intervals)
☐	☒	For null hypothesis testing, the test statistic (e.g. *F*, *t*, *r*) with confidence intervals, effect sizes, degrees of freedom and *P* value noted<br>*Give P values as exact values whenever suitable.*
☒	☐	For Bayesian analysis, information on the choice of priors and Markov chain Monte Carlo settings
☒	☐	For hierarchical and complex designs, identification of the appropriate level for tests and full reporting of outcomes
☐	☒	Estimates of effect sizes (e.g. Cohen's *d*, Pearson's *r*), indicating how they were calculated

Our web collection on statistics for biologists contains articles on many of the points above.

Software and code

Policy information about availability of computer code

Data collection: No code was used for data collection

Data analysis: The following programs were used for data analysis:
R (3.2.3, 3.6.2 and 4.0.3)
Bowtie2 (2.3.4.3)
FastQC (0.11.9)
BWA (0.7.17)
Samtools (1.2 and 1.9)
deepTools (3.5.0)
MACS2 (2.2.7.1)
deepTools (3.5.0)
clusterprofiler (3.16.1)
BD FACSDiva software (8.0.1)
longshot (0.4.1)
pysam (0.16)
htslib (1.14)
SCTransform (0.3.2)
Seurat (3.2.2)
Tidyverse (1.3.0)
Rcolorbrewer (1.1-2)
ComplexHeatmap (2.4.3)
Slingshot (1.6.1)
tradeSeq (1.2.01)

ggbeeswarm (0.6.0)
cutadapt (version 2.10)
RSamtools (2.0.3)
DESeq2 (1.24.0)
BEDOPS (2.4.39)
dplyr (2.1.1)
bedtools (2.27.1)
Minimac (version 3)
IMPUTE2 (version 2.2.2)
SNPTEST (version 2.2)
LDassoc

Custom computer code used in this study is available at 10.5281/zenodo.6335339.

For manuscripts utilizing custom algorithms or software that are central to the research but not yet described in published literature, software must be made available to editors and reviewers. We strongly encourage code deposition in a community repository (e.g. GitHub). See the Nature Research guidelines for submitting code & software for further information.

Data

Policy information about availability of data

All manuscripts must include a data availability statement. This statement should provide the following information, where applicable:

- Accession codes, unique identifiers, or web links for publicly available datasets
- A list of figures that have associated raw data
- A description of any restrictions on data availability

All ATAC-seq, RNA-seq and ChIP-seq data generated within this project have been uploaded into the Gene Expression Omnibus under the access code GSE163001 with the subseries GSE162948, GSE163000, GSE163485 and GSE163487. The mass spectrometry proteomics data have been deposited to the ProteomeXchange Consortium via the PRIDE90 partner repository with the dataset identifier PXD029522. Human RNA-Seq data for different tissue types were downloaded from the TCGA data portal (https://tcga-data.nci.nih.gov/) and from the GTex portal (https://gtexportal.org/). TCGA ATAC-seq normalised count data were downloaded from https://gdc.cancer.gov/about-data/publications/ATACseq-AWG. Normalised DNA accessibility signal data were downloaded from https://zenodo.org/record/3838751#.YJgMJC2ZM0o. Molecular signature data were downloaded from the Molecular Signature Database (MSigDB version 7.1.1) (http://www.gsea-msigdb.org/gsea/msigdb/). Protein interaction data were obtained from the STRING database version 11.0 (https://string-db.org). CRISPR-Cas9 screen CERES scores were downloaded from https://portals.broadinstitute.org/achilles. RCC GWAS meta-analysis summary data were provided by M.P.P. (purduem@mail.nih.gov) and S.J.C. (chanocks@mail.nih.gov) and they are available in the Supplementary Tables 7-8. Data from the original GWAS studies that comprise the meta-analysis data set are available either from dbGaP (NCI-1, accession number phs000351.v1.p1; NCI-2, phs001736.v1.p1; IARC-2, phs001271.v1.p1) or from the investigators upon reasonable request (IARC-1, Paul Brennan, brennanp@iarc.fr; MDA, Jian Gu, jiangu@mdanderson.org). Other data that support the findings of this study are available from the corresponding author upon reasonable request.

Field-specific reporting

Please select the one below that is the best fit for your research. If you are not sure, read the appropriate sections before making your selection.

☒ Life sciences ☐ Behavioural & social sciences ☐ Ecological, evolutionary & environmental sciences

For a reference copy of the document with all sections, see nature.com/documents/nr-reporting-summary-flat.pdf

Life sciences study design

All studies must disclose on these points even when the disclosure is negative.

Sample size	No statistical method was used to predetermine sample size. For key in vitro experiments, a minimum of three independent experiments were performed, with further validation in additional model cell lines. For in vivo experiments a minimum of 6 tumours/group was used, with further validation obtained from additional genetic constructs and/or cell lines. The sample sizes were determined based on previous experience from similar experiments.
Data exclusions	No data were excluded from the analyses.
Replication	The reproducibility of the experimental findings were verified by performing additional independent experiments or by having several technical replicates (as described in the figure legends). Furthermore, independent experiments were also conducted in several cell lines to ensure the findings were reproducible as well as cross-checking the findings with clinical data. All attempts at replication were confirmed to be successful.
Randomization	Samples were not randomized. For mouse experiments mice were age and sex matched to reduce variability between groups and all experimental groups were analyzed in parallel. For in vitro experiments, the conditions were controlled as well as possible to reduce unintended variability and the need for randomization.
Blinding	The experimental groups were not blinded. For most in vitro experiments blinding is not feasible, but whenever possible, data collection was performed automatically and/or with internal controls, reducing the need for blinding. Similarly, for mouse experiments tumour growth was tracked by caliper measurements with additional validation by bioluminescence imaging, reducing the need for blinding.

Reporting for specific materials, systems and methods

We require information from authors about some types of materials, experimental systems and methods used in many studies. Here, indicate whether each material, system or method listed is relevant to your study. If you are not sure if a list item applies to your research, read the appropriate section before selecting a response.

Materials & experimental systems

n/a	Involved in the study
☐	☒ Antibodies
☐	☒ Eukaryotic cell lines
☒	☐ Palaeontology and archaeology
☐	☒ Animals and other organisms
☐	☒ Human research participants
☒	☐ Clinical data
☒	☐ Dual use research of concern

Methods

n/a	Involved in the study
☐	☒ ChIP-seq
☐	☒ Flow cytometry
☒	☐ MRI-based neuroimaging

Antibodies

Antibodies used

For western blotting: PAX8 (Santa Cruz Biotech, sc-81353 1:250), HNF1B (Human Protein Atlas, HPA002083 1:5000), MYC (Abcam, ab32072, 1:1000), HIF2A (Novus Biologicals, NB100-122, 1:1000), VHL (BD Pharmingen, 565183, 1:1000), CCND1 (Abcam, ab134175, 1:1000), HIF1A (R&D systems, MAB1536, 1:500), and B-actin (Sigma-Aldrich, A1978, 1:20000) antibodies. Secondary antibodies were polyclonal goat anti-mouse IgG/HRP (Dako, P0447, 1:10000) and polyclonal goat anti-rabbit IgG/HRP conjugated (Dako, P0448, 1:5000).

For IHC: Human Vimentin (Cell Signaling Technology; cat. 5741, 1:100), HIF2A (Santa Cruz sc-46691,1:200), PAX8 primary antibody clone MRQ-50 (363M-16, Cell Marque) and the HNF1B primary antibody (Human Protein Atlas, HPA002083)

For co-IP: PAX8 (ProteinTech 10336-1-AP), HIF2A (Abcam, ab199), rabbit polyclonal IgG (Abcam, ab27478) and HIF1B (Santa Cruz Biotech, H-10, sc-55526, 1:200). Secondary antibodies used were anti-mouse IgG/HRP for IP (Abcam, ab131368, 1:5000) and VeriBlot for IP detection (HRP) (Abcam, ab131366, 1:5000)

Validation

1. Polyclonal goat anti-mouse IgG/HRP (Dako, P0447, 1:10000) - antibody technical datasheet [https://www.agilent.com/cs/ library/packageinsert/public/104706002.PDF]
2. Polyclonal goat anti-rabbit IgG/HRP conjugated (Dako, P0448, 1:5000) - antibody technical datasheet [https:// www.agilent.com/cs/library/packageinsert/public/104707002.PDF]
3. B-actin (Sigma-Aldrich, A1978, 1:20000) - antibody technical datasheet [https://www.sigmaaldrich.com/content/dam/sigma-aldrich/docs/Sigma/Datasheet/6/a1978dat.pdf]
4. HIF2A (Novus Biologicals, NB100-122, 1:1000) - antibody technical datasheet [https://www.novusbio.com/PDFs/ NB100-122.pdf].
5. VHL (BD Biosciences, 564183, 1:1000) - antibody technical datasheet [http://www.bdbiosciences.com/ds/pm/tds/564183.pdf]
6. PAX8 (Santa Cruz Biotech, sc-81353 1:250)-antibody technical datasheet [https://datasheets.scbt.com/sc-81353.pdf]
7. HNF1B (Human Protein Atlas, HPA002083 1:5000)-antibody technical datasheet [https://www.atlasantibodies.com/api/print_datasheet/HPA002083.pdf]
8. MYC (Abcam, ab32072, 1:1000)-antibody technical datasheet [https://www.abcam.com/c-myc-antibody-y69-bsa-and-azide-free-ab168727.pdf]
9. HIF1B (Santa Cruz Biotech, H-10, 1:200)-antibody technical datasheet [https://datasheets.scbt.com/sc-55526.pdf]
10. HIF1A (R&D systems, MAB1536, 1:500), -antibody technical datasheet [https://resources.rndsystems.com/pdfs/datasheets/mab1536.pdf?v=20211102&_ga=2.202325457.1710034994.1635864392-808840216.1615813694]
11. CCND1 (Abcam, ab134175, 1:1000) – antibody technical datasheet [https://www.abcam.com/cyclin-d1-antibody-epr2241-c-terminal-ab134175.pdf]
12. Human Vimentin (Cell Signaling Technology; cat. 5741, 1:100) - antibody technical datasheet [https://www.cellsignal.com/datasheet.jsp?productId=5741&images=1]
13. HIF2A (Santa Cruz sc-46691,1:200) – antibody technical datasheet [https://datasheets.scbt.com/sc-46691.pdf]
14. PAX8 primary antibody clone MRQ-50 (363M-16, Cell Marque) - antibody technical datasheet [https://www.cellmarque.com/antibodies/CM/2127/PAX-8_MRQ-50]
15. HIF2A (Abcam, ab199) – antibody technical datasheet [https://www.abcam.com/hif-2-alpha-antibody-ab199.pdf]
16. rabbit polyclonal IgG (Abcam, ab27478) - antibody technical datasheet [https://www.abcam.com/rabbit-igg-polyclonal-isotype-control-ab27478.pdf]
17. anti-mouse IgG/HRP for IP (Abcam, ab131368, 1:5000) - antibody technical datasheet [https://www.abcam.com/veriblot-for-ip-detection-reagent-hrp-ab131366.pdf]
18. VeriBlot for IP detection (HRP) (Abcam, ab131366, 1:5000) - antibody technical datasheet [https://www.abcam.com/mouse-igg-for-ip-hrp-ab131368.html]

Eukaryotic cell lines

Policy information about cell lines

Cell line source(s)	The UOK101 cell line was obtained from M. Linehan (the UOB Tumor Cell Line Repository, National Cancer Institute, Bethesda, MD). The HK2 cell line was obtained from C. Frezza (MRC Cancer Unit, Cambridge, UK). ACHN and CAKI-2 were obtained from E. Maher (Department of Medical Genetics, University of Cambridge, UK). All other human cancer cell lines and the HEK293T cells were obtained from J. Massagué (MSKCC, New York, USA). 786-M1A and OS-LM1 are the respective metastatic derivatives of 786-O and OS-RC2 cells and have been previously described (Vanharanta et al., Nat Med. (2013) PMID: 23223005). 2806-LM1A is a metastatic derivative of 786-O and it does not carry a luciferase reporter gene. C-M1A HIF2A-/- is a single cell- derived HIF2A-/- clone from 786-M1A cells generated by CRISPR-Cas9 mediated knockout of HIF2A.
Authentication	Cell lines were authenticated by short tandem repeat profiling.
Mycoplasma contamination	Cell lines were confirmed to be mycoplasma negative using the MycoAlertTM Mycoplasma Detection Kit (Lonza, LT07-318) or by qRT-PCR (PhoenixDx® Mycoplasma Mix).
Commonly misidentified lines (See ICLAC register)	At the time of the study, none of the cell lines used in this study were listed in the database of commonly misidentified cell lines maintained by ICLAC.

Animals and other organisms

Policy information about studies involving animals; ARRIVE guidelines recommended for reporting animal research

Laboratory animals	Athymic nude mice, female, 5-8 weeks old (Charles River Laboratories 490 (Homozygous)). NSG mice, male, 5-7 weeks old (Charles River Laboratories, strain: NOD.Cg-Prkdcscid Il2rgtm1Wjl/SzJ). The housing conditions were as follows: 12/12h dark/light cycle, humidity 45-65%, temperature 20-24°C.
Wild animals	This study did not involve wild animals.
Field-collected samples	This study did not involve field-collected samples.
Ethics oversight	Home Office (UK) and the University of Cambridge Animal Welfare and Ethical Review Body.

Note that full information on the approval of the study protocol must also be provided in the manuscript.

Human research participants

Policy information about studies involving human research participants

Population characteristics	A tissue microarray with 427 ccRCC patients (170 females, 257 males, age range 28-90 with a median of 65) tumours represented on it from the full range of stages of disease was used. It was created as previously published (Laird A, O'Mahony FC, Nanda J, Riddick AC, O'Donnell M, Harrison DJ, et al. Differential expression of prognostic proteomic markers in primary tumour, venous tumour thrombus and metastatic renal cell cancer tissue and correlation with patient outcome. PLoS One. 2013;8(4):e60483. doi:10.1371/journal.pone.0060483.) Normal human kidney tissue used for organoid derivation was sampled from a 75-year-old male.
Recruitment	Patients who had primary renal cell carcinoma at the time of surgery or at a later date were identified from a prospectively compiled database. Formalin fixed paraffin embedded (FFPE) tumour samples were identified from 427 of these patients who underwent radical nephrectomy between 1983 and 2010, in the Department of Urology, Edinburgh. Where possible, written informed consent was gained for use of tissue surplus to diagnostic requirement and linked anonymised patient data. Normal human kidney tissue from a nephrectomy specimen was sampled with informed consent.
Ethics oversight	Ethical approval to use these archived tissues was granted by the Lothian Regional Ethics Committee (08/S1101/41 and 10/S1402/33). Normal human kidney tissue used for organoid derivation was collected under an ethical approval by the East of England - Cambridge Central Research Ethics Committee (19/EE/0161).

Note that full information on the approval of the study protocol must also be provided in the manuscript.

ChIP-seq

Data deposition

☒ Confirm that both raw and final processed data have been deposited in a public database such as GEO.

☒ Confirm that you have deposited or provided access to graph files (e.g. BED files) for the called peaks.

Data access links *May remain private before publication.*	https://www.ncbi.nlm.nih.gov/geo/query/acc.cgi?acc=GSE163487

Files in database submission

SLX-14864.A002.HVL5LBBXX.s_5.r_1_trim_unmapped_merged.bl.bw
SLX-14864.A006.HVL5LBBXX.s_5.r_1_trim_unmapped_merged.bl.bw
SLX-14864.A007.HVL5LBBXX.s_5.r_1_trim_unmapped_merged.bl.bw
SLX-16309.A002.HWMMGBBXX.s_3.r_1_trim_unmapped.bl.bw
SLX-16309.A014.HWMMGBBXX.s_3.r_1_trim_unmapped.bl.bw
SLX-16309.A016.HWMMGBBXX.s_3.r_1_trim_unmapped.bl.bw
SLX-16309.A019.HWMMGBBXX.s_3.r_1_trim_unmapped.bl.bw
SLX-14864.A013.HVL5LBBXX.s_5.r_1_trim_unmapped_merged.bl.bw
SLX-14864.A014.HVL5LBBXX.s_5.r_1_trim_unmapped_merged.bl.bw
SLX-14864.A015.HVL5LBBXX.s_5.r_1_trim_unmapped_merged.bl.bw
SLX-16309.A004.HWMMGBBXX.s_3.r_1_trim_unmapped.bl.bw
SLX-16309.A007.HWMMGBBXX.s_3.r_1_trim_unmapped.bl.bw
SLX-16309.A013.HWMMGBBXX.s_3.r_1_trim_unmapped.bl.bw
SLX-16301.A002.HWCFNBBXX.s_8.r_1_trim_unmapped.bl.bw
SLX-16301.A004.HWCFNBBXX.s_8.r_1_trim_unmapped.bl.bw
SLX-16301.A005.HWCFNBBXX.s_8.r_1_trim_unmapped.bl.bw
SLX-16301.A013.HWCFNBBXX.s_8.r_1_trim_unmapped.bl.bw
SLX-16301.A014.HWCFNBBXX.s_8.r_1_trim_unmapped.bl.bw
SLX-16301.A015.HWCFNBBXX.s_8.r_1_trim_unmapped.bl.bw
SLX-16301.A006.HWCFNBBXX.s_8.r_1_trim_unmapped.bl.bw
SLX-16301.A007.HWCFNBBXX.s_8.r_1_trim_unmapped.bl.bw
SLX-16302.A007.H23WKBBXY.s_3.r_1_trim_unmapped.bl.bw
SLX-16301.A016.HWCFNBBXX.s_8.r_1_trim_unmapped.bl.bw
SLX-16301.A018.HWCFNBBXX.s_8.r_1_trim_unmapped.bl.bw
SLX-16301.A019.HWCFNBBXX.s_8.r_1_trim_unmapped.bl.bw
SLX-16302.A012.H23WKBBXY.s_3.r_1_trim_unmapped.bl.bw
SLX-16310.A002.H23WKBBXY.s_5.r_1_trim_unmapped.bl.bw
SLX-16310.A004.H23WKBBXY.s_5.r_1_trim_unmapped.bl.bw
SLX-16310.A005.H23WKBBXY.s_5.r_1_trim_unmapped.bl.bw
SLX-16310.A012.H23WKBBXY.s_5.r_1_trim_unmapped.bl.bw
SLX-16310.A013.H23WKBBXY.s_5.r_1_trim_unmapped.bl.bw
SLX-16310.A014.H23WKBBXY.s_5.r_1_trim_unmapped.bl.bw
SLX-16310.A006.H23WKBBXY.s_5.r_1_trim_unmapped.bl.bw
SLX-16310.A007.H23WKBBXY.s_5.r_1_trim_unmapped.bl.bw
SLX-16310.A019.H23WKBBXY.s_5.r_1_trim_unmapped.bl.bw
SLX-16310.A015.H23WKBBXY.s_5.r_1_trim_unmapped.bl.bw
SLX-16310.A016.H23WKBBXY.s_5.r_1_trim_unmapped.bl.bw
SLX-16310.A018.H23WKBBXY.s_5.r_1_trim_unmapped.bl.bw
SLX-14864.A002.HVL5LBBXX.s_5.r_1.fq.gz
SLX-14864.A006.HVL5LBBXX.s_5.r_1.fq.gz
SLX-14864.A007.HVL5LBBXX.s_5.r_1.fq.gz
SLX-16309.A002.HWMMGBBXX.s_3.r_1.fq.gz
SLX-16309.A014.HWMMGBBXX.s_3.r_1.fq.gz
SLX-16309.A016.HWMMGBBXX.s_3.r_1.fq.gz
SLX-16309.A019.HWMMGBBXX.s_3.r_1.fq.gz
SLX-14864.A013.HVL5LBBXX.s_5.r_1.fq.gz
SLX-14864.A014.HVL5LBBXX.s_5.r_1.fq.gz
SLX-14864.A015.HVL5LBBXX.s_5.r_1.fq.gz
SLX-16309.A004.HWMMGBBXX.s_3.r_1.fq.gz
SLX-16309.A007.HWMMGBBXX.s_3.r_1.fq.gz
SLX-16309.A013.HWMMGBBXX.s_3.r_1.fq.gz
SLX-16301.A002.HWCFNBBXX.s_8.r_1.fq.gz
SLX-16301.A004.HWCFNBBXX.s_8.r_1.fq.gz
SLX-16301.A005.HWCFNBBXX.s_8.r_1.fq.gz
SLX-16301.A013.HWCFNBBXX.s_8.r_1.fq.gz
SLX-16301.A014.HWCFNBBXX.s_8.r_1.fq.gz
SLX-16301.A015.HWCFNBBXX.s_8.r_1.fq.gz
SLX-16301.A006.HWCFNBBXX.s_8.r_1.fq.gz
SLX-16301.A007.HWCFNBBXX.s_8.r_1.fq.gz
SLX-16302.A007.H23WKBBXY.s_3.r_1.fq.gz
SLX-16301.A016.HWCFNBBXX.s_8.r_1.fq.gz
SLX-16301.A018.HWCFNBBXX.s_8.r_1.fq.gz
SLX-16301.A019.HWCFNBBXX.s_8.r_1.fq.gz
SLX-16302.A012.H23WKBBXY.s_3.r_1.fq.gz
SLX-16310.A002.H23WKBBXY.s_5.r_1.fq.gz
SLX-16310.A004.H23WKBBXY.s_5.r_1.fq.gz
SLX-16310.A005.H23WKBBXY.s_5.r_1.fq.gz
SLX-16310.A012.H23WKBBXY.s_5.r_1.fq.gz
SLX-16310.A013.H23WKBBXY.s_5.r_1.fq.gz
SLX-16310.A014.H23WKBBXY.s_5.r_1.fq.gz
SLX-16310.A006.H23WKBBXY.s_5.r_1.fq.gz
SLX-16310.A007.H23WKBBXY.s_5.r_1.fq.gz
SLX-16310.A019.H23WKBBXY.s_5.r_1.fq.gz
SLX-16310.A015.H23WKBBXY.s_5.r_1.fq.gz
SLX-16310.A016.H23WKBBXY.s_5.r_1.fq.gz

	SLX-16310.A018.H23WKBBXY.s_5.r_1.fq.gz<br>SLX-14864.A002.HFH52BBXY.s_1.r_1.fq.gz<br>SLX-14864.A006.HFH52BBXY.s_1.r_1.fq.gz<br>SLX-14864.A007.HFH52BBXY.s_1.r_1.fq.gz<br>SLX-14864.A013.HFH52BBXY.s_1.r_1.fq.gz<br>SLX-14864.A014.HFH52BBXY.s_1.r_1.fq.gz<br>SLX-14864.A015.HFH52BBXY.s_1.r_1.fq.gz
Genome browser session<br>(e.g. UCSC)	NA

Methodology

Replicates

HIF2A M1A ChIP: 3 biological replicates
HIF2A M1A input: 4 biological replicates
HIF2A LM1B ChIP: 3 biological replicates
HIF2A LM1B input: 3 biological replicates

PAX8 M1A ChIP: 3 biological replicates
PAX8 M1A input: 3 biological replicates
PAX8 LM1B ChIP: 3 biological replicates
PAX8 LM1B ChIP: 4 biological replicates

HNF1B M1A ChIP: 3 biological replicates
HNF1B M1A input: 3 biological replicates
HNF1B LM1B ChIP: 3 biological replicates
HNF1B LM1A input: 3 biological replicates

Sequencing depth

All the samples are single-end with 50bp read length.

The total number of reads (after excluding genes mapping to mouse genome) and the number of reads after filtering out reads with mapping quality < 20, reads mapping to blacklisted regions and reads mapping to regions other than chr1 to 22, X and Y is reported for each sample.

SLX-14864.A002 Total: 24278880; After filtering: 22016100
SLX-14864.A006 Total: 16141095; After filtering: 14547194
SLX-14864.A007 Total: 17380560; After filtering: 15697922
SLX-16309.A002 Total: 10940226; After filtering: 9915172
SLX-16309.A014 Total: 14594845; After filtering: 13162705
SLX-16309.A016 Total: 14776625; After filtering: 13337506
SLX-16309.A019 Total: 10291446; After filtering: 9284789
SLX-14864.A013 Total: 13906559; After filtering: 12505916
SLX-14864.A014 Total: 87053321; After filtering: 79063102
SLX-14864.A015 Total: 18828982; After filtering: 17082291
SLX-16309.A004 Total: 51148025; After filtering: 46215659
SLX-16309.A007 Total: 16635642; After filtering: 14945684
SLX-16309.A013 Total: 30450304; After filtering: 27511220
SLX-16301.A002 Total: 11127778; After filtering: 10111323
SLX-16301.A004 Total: 27172651; After filtering: 24631106
SLX-16301.A005 Total: 20029312; After filtering: 18224040
SLX-16301.A013 Total: 22492314; After filtering: 20283038
SLX-16301.A014 Total: 39913582; After filtering: 35968614
SLX-16301.A015 Total: 16962961; After filtering: 15292985
SLX-16301.A006 Total: 21505330; After filtering: 19517946
SLX-16301.A007 Total: 24900285; After filtering: 22550039
SLX-16302.A007 Total: 10405114; After filtering: 9444550
SLX-16301.A016 Total: 29484377; After filtering: 26557099
SLX-16301.A018 Total: 29616225; After filtering: 26685369
SLX-16301.A019 Total: 22889998; After filtering: 20535788
SLX-16302.A012 Total: 4513129; After filtering: 4075220
SLX-16310.A002 Total: 21338846; After filtering: 19156581
SLX-16310.A004 Total: 5656074; After filtering: 5189524
SLX-16310.A005 Total: 18598964; After filtering: 17006151
SLX-16310.A012 Total: 26730720; After filtering: 24030433
SLX-16310.A013 Total: 29122467; After filtering: 26298413
SLX-16310.A014 Total: 36061004; After filtering: 32708244
SLX-16310.A006 Total: 24232763; After filtering: 21942289
SLX-16310.A007 Total: 10971401; After filtering: 9941291
SLX-16310.A019 Total: 13340589; After filtering: 12128048
SLX-16310.A015 Total: 28101167; After filtering: 25312242
SLX-16310.A016 Total: 15663471; After filtering: 14116857
SLX-16310.A018 Total: 9664508; After filtering: 8725758

Antibodies

PAX8 (ProteinTech 10336-1-AP), HNF1B (Human Protein Atlas, HPA002083), HIF2A (Novus Biologicals NB100-122) and rabbit polyclonal IgG (Abcam, ab27478)

Peak calling parameters

Mapping
bwa mem -M hg38.fa chip.fq > chip.sam
samtools view -S -b -h -T hg38.fa chip.sam | samtools sort -O bam -T chip.tmp -o chip.bam;
samtools index chip.bam

Peak calling
macs2 callpeak --bdg -t chip.bam -c input.bam -f BAM -g 2913022398

Data quality

Number of peaks with fold change more than 5 and FDR 0.05

HIF2A_M1A_1: 1541
HIF2A_M1A_2: 276
HIF2A_M1A_3: 649
HIF2A_LM1B_1: 541
HIF2A_LM1B_2: 1766
HIF2A_LM1B_3: 748

HNF1B_M1A_1: 2522
HNF1B_M1A_2: 7511
HNF1B_M1A_3: 5360
HNF1B_LM1B_1: 3210
HNF1B_LM1B_2: 6435
HNF1B_LM1B_3: 5310

PAX8_M1A_1: 3116
PAX8_M1A_2: 1044
PAX8_M1A_3: 6930
PAX8_LM1B_1: 6665
PAX8_LM1B_2: 4045
PAX8_LM1B_3: 16225

Software

FastQC (version 0.11.7)
Cutadapt(version 1.10.0)
Bowtie 2 (version 2.3.4.3)
MACS2 (version 2.2.7.1)
Samtools (version 1.2)
BEDOPS (version 2.4.37)
deepTools (version 3.5.0)
bedtools (version 2.27.1)

Flow Cytometry

Plots

Confirm that:

☒ The axis labels state the marker and fluorochrome used (e.g. CD4-FITC).

☒ The axis scales are clearly visible. Include numbers along axes only for bottom left plot of group (a 'group' is an analysis of identical markers).

☒ All plots are contour plots with outliers or pseudocolor plots.

☒ A numerical value for number of cells or percentage (with statistics) is provided.

Methodology

Sample preparation

In brief, the cells were either lentivirally transduced with mCherry, GFP or BFP sgRNA/shRNA expression vectors, mixed and plated onto multi-well plates in triplicate. For flow cytometry analyses at the different time points, the mixed cells populations were trypsinized and directly analyzed on the instrument specified below.
Source of cells : human renal cancer cell lines

Instrument

LSR Fortessa (BD Biosciences)

Software

BD FACSDiva software (8.0.1)

Cell population abundance

The abundance of relevant cell population was determined based on the specific fluorescent marker expressed by the cells. The control cell population increased over times and outgrew the knockout cell population that resulted in reduced percentage/ abundance of the knockout cell population.

Gating strategy

Competitive proliferation assay gating strategy
1. FSC-A / SSC-A : to select for live cell population
2. FSC-W / SSC-A : to select for single cells
3. mCherry (561nm/610nm), BFP (383nm/445nm) or GFP (488nm/510nm) : to discriminate between the cell populations

☒ Tick this box to confirm that a figure exemplifying the gating strategy is provided in the Supplementary Information.
